# A meta-analysis of graft survival, patient survival and delayed graft function in first-time and repeat kidney transplants

**DOI:** 10.1093/ndt/gfaf066

**Published:** 2025-04-15

**Authors:** Mehmet Kanbay, Sama Mahmoud Abdel-Rahman, Crischentian Brinza, Lasin Ozbek, Elif Yayci, Ozgur Aktas, Candan Genc, Mustafa Guldan, Ezgi N Alper, Alexandru Burlacu, Andreea Covic, Adrian Covic

**Affiliations:** Department of Internal Medicine, Division of Nephrology, Koç University School of Medicine, Istanbul, Turkey; Department of Internal Medicine, Koç University, School of Medicine, Istanbul, Turkey; Faculty of Medicine, University of Medicine and Pharmacy “Grigore T Popa”, Iasi, Romania; Department of Internal Medicine, Koç University, School of Medicine, Istanbul, Turkey; Department of Internal Medicine, Koç University, School of Medicine, Istanbul, Turkey; Department of Internal Medicine, Koç University, School of Medicine, Istanbul, Turkey; Department of Internal Medicine, Koç University, School of Medicine, Istanbul, Turkey; Department of Internal Medicine, Koç University, School of Medicine, Istanbul, Turkey; Department of Internal Medicine, Koç University, School of Medicine, Istanbul, Turkey; Faculty of Medicine, University of Medicine and Pharmacy “Grigore T Popa”, Iasi, Romania; Institute of Cardiovascular Diseases “Prof. Dr George I.M. Georgescu”, Iasi, Romania; Faculty of Medicine, University of Medicine and Pharmacy “Grigore T Popa”, Iasi, Romania; Nephrology Clinic, Dialysis, and Renal Transplant Center – “C.I. Parhon” University Hospital, Iasi, Romania; Faculty of Medicine, University of Medicine and Pharmacy “Grigore T Popa”, Iasi, Romania; Nephrology Clinic, Dialysis, and Renal Transplant Center – “C.I. Parhon” University Hospital, Iasi, Romania

**Keywords:** first-time transplants, graft survival, kidney transplantation, patient survival, repeat transplants

## Abstract

**Background:**

Previous evidence showed that while first-time kidney transplants (KT) typically yield better outcomes, repeat and subsequent transplants were associated with increased risks of graft failure and adverse patient outcomes, yet conflicting findings exist. The aim of this meta-analysis is to compare graft survival and delayed graft function (DGF) outcomes in first-time KT, repeat KT (regrafts) and subsequent KT.

**Methods:**

Relevant studies were identified through comprehensive searches in PubMed, Web of Science, Cochrane Library, MEDLINE (Ovid) and Scopus until 8 October 2024. Primary outcomes include graft survival and DGF, compared with repeat and subsequent KT.

**Results:**

The meta-analysis included a total of 16 studies. Analysis on long-term graft survival revealed that patients who underwent a first KT had significantly better graft survival compared with those who received a second transplant [86.7% versus 77.6%; odds ratio (OR) 1.40, 95% confidence interval (CI) 1.14–1.71, *P* = .001]. At 5 years post-transplant, first KT recipients continued to demonstrate superior graft survival (OR 1.41, 95% CI 1.13–1.77, *P* = .003), although this difference diminished by 10 years, with no significant disparity observed (OR 1.26, 95% CI 0.88–1.81, *P* = .20). Graft survival at 5 years was also significantly higher in second KT recipients compared with those undergoing a third transplant (OR 2.66, 95% CI 1.86–3.80, *P* < .00001). Patient survival outcomes were largely comparable between first and second KT groups, with no statistically significant differences in overall survival (OR 1.25, 95% CI 0.87–1.81, *P* = .23). At specific time points, the 5-year survival rate showed a borderline non-significant trend favoring first KT recipients (OR 1.63, 95% CI 0.97–2.73, *P* = .06), while the 10-year survival rate showed no difference (OR 0.94, 95% CI 0.67–1.32, *P* = .71). Survival rates between second and subsequent retransplants (e.g. third or fourth KT) showed no significant variation, including at 5 years (*P* = .37 and *P* = .90, respectively). DGF rates did not differ significantly between first and second KT recipients (*P* = .11).

**Conclusion:**

These findings underscore the superior graft survival associated with first and second KT compared with subsequent retransplants, particularly in the early post-transplant period, while highlighting the lack of significant differences in overall patient survival across groups; however, variability in outcomes due to study heterogeneity and patient-specific factors warrants cautious interpretation and tailored clinical approaches.

## INTRODUCTION

Kidney transplantation is the optimal treatment for patients with end-stage kidney disease, offering superior survival and quality of life compared with dialysis [1]. Despite advancements in immunosuppression, surgical techniques and perioperative care, long-term graft survival remains limited, with chronic allograft failure often necessitating dialysis or retransplantation [2]. Approximately 20% of kidney transplant (KT) recipients experience allograft failure within 5 years, nearly 50% within 10 years and 50%–60% will lose their graft over their lifetime, with retransplantation accounting for up to 20% of all procedures [3–5].

Retransplantation offers a survival advantage over dialysis, even in high-risk populations such as older adults and highly sensitized patients [6–8]. While outcomes for second transplants are comparable to primary transplants, third and subsequent transplants present higher risks, including infection, malignancy, surgical complications and hyperacute rejection linked to elevated panel-reactive antibody levels [9–11]. Despite these challenges, advancements in transplant medicine continue to improve outcomes for recipients.

Although the total number of candidates added to the KT waitlist has steadily increased, the proportion of those awaiting retransplantation has declined, while the total number of failed allografts has remained steady over two decades. Since 2007, the absolute number of candidates with prior transplants has remained relatively flat, decreasing from 14% in 2004 to 10% in 2018 [4, 5]. This trend persists despite growing evidence of acceptable outcomes with second and third transplants and supportive policies that exclude retransplant candidates from restrictive survival metrics in the USA [12]. These shifts underscore the need to better understand retransplant outcomes and address barriers to equitable access, particularly among underserved populations.

This meta-analysis aims to synthesize and analyze existing data on outcomes of kidney retransplantation, focusing on patient and graft survival across primary, second, third and fourth transplants. By consolidating these findings, this meta-analysis seeks to provide insights into optimizing clinical practice and guiding allocation policies for scarce donor kidneys, particularly in the context of retransplantation.

## MATERIALS AND METHODS

### Study design and search strategy

This study is a systematic review and meta-analysis designed to compare graft survival, patient survival and delayed graft function (DGF) across first-time KT, repeat KT (regrafts) and subsequent KT. The protocol for this meta-analysis was registered with the PROSPERO database (CRD42025595131). The study was conducted following the Preferred Reporting Items for Systematic Reviews and Meta-Analyses (PRISMA) guidelines. Relevant studies published until 1 December 2024 were identified through searches in PubMed, Web of Science, Cochrane Library, Embase and Scopus. Keywords and Medical Subject Headings (MeSH) terms combined in the search included “kidney transplant,” “graft survival,” “patient survival,” “delayed graft function,” “repeat transplant,” “regraft” and “subsequent kidney transplant.” The detailed search strategy is shown in Supplementary data, Table S1. Only peer-reviewed full-text articles published in English that met predefined eligibility criteria were included in the analysis.

**Table 1:** Baseline characteristics of the included studies.

Study	Study design	Follow-up duration	Study population	Baseline characteristics	Outcome measures	Key findings
Benko (2019) [25]	Retrospective cohort	5 years	Total (*n* = 216): - Group 2 (*n* = 108): 2nd KT - Group 3+ (*n* = 108): 3rd, 4th, 5th KT	Group 2 (*n* = 108): Sex: 58 males (53.7%) Age: mean 43.9 years BMI: mean 25.7 kg/m^2^ Group 3+ (*n* = 108): Sex: 57 males (52.8%) Age: mean 43.2 years BMI: mean 24.2 kg/m^2^	Surgical complication and ICU stay DGF GS PS	Comparable surgical complications, ICU stays, DGF, GS and PS in 2nd vs >3 KT Previous transplantation and biopsy proven rejection are predictors of GS in >3 KT
Dabare (2019) [13]	Retrospective cohort	Median of 79 months	Total (2561) - 1st KT: *n* = 2154 - 2nd KT: *n* = 330 - 3rd/4th KT: *n* = 77	1st KT (*n* = 2154): Age: mean 47.8 years Sex: 1366 males (63%) No mismatches: 2072 (96%) 2nd KT (*n* = 330): Age: mean 42.6 years Sex: 183 males (55%) No mismatches: 309 (94%) 3rd/4th KT (*n* = 77): Age: mean 43.8 years Sex: 33 males (43%) No mismatches: 60 (78%)	GS PS DCGS	PS was indifferent in graft number DCGS shorter with multiple KT
Ehrsam (2022) [26]	Retrospective cohort	10 years	Total (*n* = 1598): - 1st KT: *n* = 1376 - 2nd KT: *n* = 222	1st KT (*n* = 1376) : Age: mean 48.4 years Sex: 63.2% male HLA mismatches: median 3 2nd KT (*n* = 222): Age: mean 46.2 years Sex: 62.5% male HLA mismatches: median 4	GF PS DCGS	Comparable GS within 10 years after KT In multiple Cox regression model, 2nd KT is risk factor for GS Causes and rate of mortality similar in first vs second KT
Fukuhara (2023) [14]	Retrospective cohort	Mean: 51 months	Total (*n* = 898): - 1st KT: *n* = 865 - 2nd KT: *n* = 33	1st KT (*n* = 865): Age: mean 50 years Sex: 551 males (63.7%) BMI: mean 22.2 kg/m^2^ 2nd KT (*n* = 33): Age: mean 50 years Sex: 22 males (63.6%) BMI: mean 20.7 kg/m^2^	GS DCGS PS	No significant difference in DCGS and PS between 1st KT vs 2nd KT Rejection was most common cause of graft loss Infection was most common cause of mortality in both groups
Han (2019) [16]	Retrospective cohort	Mean duration: 1st KT: 75.2 months 2nd KT: 84.5 months 3rd KT: 79.5 months	Total (*n* = 2556): - 1st KT: *n* = 2337 - 2nd KT: *n* = 201 - 3rd KT: *n* = 18	1st KT (*n* = 2337): Age: mean 42.3 years Sex: 1416 males (60.6%) BMI: mean 22.45 kg/m^2^ Mean HLA mismatch: 3.3 2nd KT (*n* = 201): Age: mean 45.3 years Sex: 127 males (63.2%) BMI: mean 21.33 kg/m^2^ Mean HLA mismatch: 3.3	GS DCGS PS	GS, DCGS and PS similar in 1st KT, 2nd KT and 3rd KT Prognostic factors for patient and GS were older age, male sex and immunosuppressants
Heldal (2017) [17]	Retrospective cohort	Recruitment in 2000–2014, last survival data obtained in 2015	KT patients older than 65 years (*n* = 733): - 1st KT: *n* = 687 - 2nd KT: *n* = 46	1st KT (*n* = 687): Age: mean 71.0 years Sex: 492 males (71.6%) 2nd KT (*n* = 46): Age: mean 69.3 years Sex: 28 males (61%)	GS DCGS PS	GS and PS was similar Death with functioning graft loss is the most common cause of graft loss in both groups Recipient age, donor age and time on dialysis are independent risk factors for GS
Izquierdo (2010) [24]	Prospective cohort	57.4 months	Total (*n* = 82): - 3rd KT: *n* = 74 - 4th KT: *n* = 8	Total (*n* = 82): Sex: 49 males (59.8%) Mean time on dialysis: 126.89 months	GS PS	GS is lower in 4th KT compared with 3rd KT Comparable PS between 3rd vs 4th KT
Khubutiya (2021) [18]	Retrospective cohort	NA	Total (*n* = 339) 1st KT: *n* = 245 2nd KT: *n* = 94	Total (*n* = 339) Age: median 45 years Sex: 194 males (57.2%) BMI: median 24.5 kg/m^2^	GS PS	2nd KT patients with expanded criteria donors have lower 5-year PS and GS rate than 1st KT patients with expanded criteria donors GS was better in 1st KT compared with 2nd KT
Kim (2017) [19]	Retrospective cohort	Mean: 82.5 months	KT patients with prior IgA nephropathy (*n* = 88) - 2nd KT: *n* = 7	Total (*n* = 88) Age: mean 38.05 years Sex: 52 males (59.1%) >3 HLA mismatch: 29 (33%) 2nd KT: 7	Recurrence of IgAN DGF GS PS	5- and 10-year GS and PS rates were 100% in transplantation group
Oh (2022) [9]	Retrospective cohort	20 years	KT from living donors (*n* = 1429) 1st KT: *n* = 1335 2nd KT: *n* = 74	1st KT (*n* = 1335) Age: mean 45 years Sex: 791 males (58.0%) BMI: mean 23 kg/m^2^ 2nd KT (*n* = 74) Age: mean 47 years Sex: 37 males (50.0%) BMI: mean 22 kg/m^2^	GS DCGS PS Graft function by sCr for 10 years	Comparable GS, PS rates and graft function in 1st vs 2nd KT Age of donor and number of HLA II mismatches associate with GS Age, hypertension, number of HLA II mismatches associate with PS No difference in malignancy risk
Ooms (2015) [20]	Retrospective case–control	Case–control 1st KT: Median 58 months 2nd KT in ipsilateral fossa: Median 60 months	Total (*n* = 396) - Case–control 1st KT: *n* = 297 - 2nd KT in ipsilateral fossa: *n* = 99	Case–control 1st KT (*n* = 297): Age: 45 years Sex: 165 males (56%) BMI: mean 24 kg/m^2^ HLA mismatches: median 3.0 2nd KT in ipsilateral fossa: (*n* = 99): Age: mean 43 years Sex: 55 males (56%) BMI: mean 24 kg/m^2^ HLA mismatches: median 2.0	Vascular complication GS PS Renal function	Higher vascular complications and rate of graft nephrectomy in 2nd KT 3- and 12- month posttransplantation GFR was lower in 2nd KT Similar 1 to 10 year GS and PS rates in control and 2nd KT Lower GS in 1st month of transplantation in 2nd KT compared with controls
Pardinhas (2022) [11]	Retrospective longitudinal cohort	Mean: 63.9 months	Total (*n* = 499) - Group 1 (*n* = 13): 2nd KT and ≥ 60 years old - Group 2 (*n* = 96): 2nd KT and <60 years old and retransplant - Group 3 (*n* = 390): 1st KT and ≥60 years old	Group 1 (*n* = 13): Age: mean 62.85 years Sex: 13 males (100%) BMI: mean 25.03 kg/m^2^ HLA mismatches: mean 4.08 Group 2 (*n* = 96): Age: mean 40.44 years Sex: 57 males (59.4%) BMI: mean 22.5 kg/m^2^ HLA mismatches: mean 3.61 Group 3 (*n* = 390): Age: mean 64.5 years Sex: 280 males (71.8%) BMI: – HLA mismatches: mean 3.9	Acute rejection DGF GS DCGS PS	Comparable DGF, DCGS, PS Lower serum Cr l in older 1st KT patients Doubled mortality rate in Group 3 although not significant
Roozbeh (2018) [22]	Retrospective cohort	Mean: 57.2 months	Total (*n* = 268) - 1st KT: *n* = 200 - 2nd KT: *n* = 68	1st KT (*n* = 200): Age: mean 56.0 years Sex: 140 males (70.0%) 2nd KT (*n* = 68): Age: mean 35.6 years Sex: 46 males (67.6%)	GS Acute rejection DGF PS	Longer hospital stay after KT in 2nd KT Similar PS and GS Lower GS in DGF and acute rejection
Silva (2022) [23]	Retrospective observational	At least 1 year	Total (*n* = 81) - 1st KT: *n* = 50 - 2nd KT: *n* = 31	1st KT (*n* = 50): Age: mean 43.84 years Sex: 25 males (50%) BMI: mean 23.57 kg/m^2^ 2nd KT (*n* = 31): Age: mean 44.87 years Sex: 20 males (64.52%) BMI: mean 22.59 kg/m^2^	DGF Kidney function (sCr) Infection and rejection episodes GS PS	Significantly lower GS in 2nd KT More frequent rejection episodes in 2nd KT Similar PS between 1st and 2nd KT
Telkes (2021) [10]	Retrospective, observational	Recruitment in 2011–2016, end of observation 2019	Total (*n* = 666) - 1st KT: *n* = 646 - 3rd KT: *n* = 20	1st KT (*n* = 646): Age: mean 53.4years Sex: males 59.9% BMI: mean 26.4 kg/m^2^ 3rd KT (*n* = 20): Age: mean 47.3 years Sex: males 70.0% BMI: mean 25.0 kg/m^2^	DGF Kidney function GS PS	Frequent DGF in 3rd KT Higher 1- to 5-year PS and GS rates in 1st KT compared with 3rd KT Moderately higher kidney function in 1st KT compared with 3rd KT

DCGS, death-censored graft survival; GS, graft survival; PS, patient survival; NA, not applicable; ICU, intensive care unit; BMI, body mass index; GFR, glomerular filtration rate; IgA, immunoglobulin A.

### Eligibility criteria

The PICOTS framework was utilized to define the eligibility criteria. Regarding Population (P), studies were included if they reported outcomes on KT recipients, specifically comparing first-time, repeat and subsequent KT. For the Intervention (I), the focus was on patients undergoing second or subsequent KT, while the Comparator (C) included first-time KT recipients. The Outcomes (O) assessed included data on graft survival (at least 1-year follow-up), patient survival (1-year, 5-year and long-term follow-up), and DGF. For Timing (T), studies were required to provide a minimum of 1-year follow-up data on graft and patient survival to be eligible for inclusion. The Setting (S) included transplant centers, healthcare institutions where kidney transplantation procedures and follow-ups were conducted. Observational studies, randomized controlled trials, cohort studies and case–control studies were eligible, provided they reported graft failure, patient mortality and the incidence of DGF. Studies were excluded if they had insufficient data or incomplete outcome reporting, were case reports, reviews or meta-analyses, or were focused solely on pediatric populations, as their outcomes might differ significantly from adults.

### Study selection

**Figure 1:**
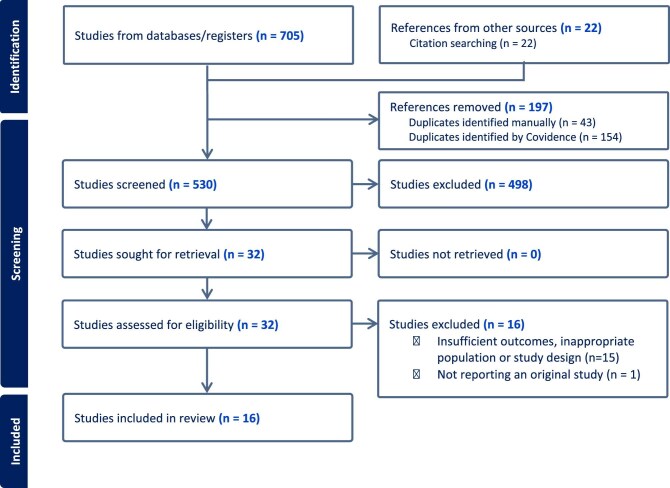
Flow diagram of the study selection process.

Two reviewers performed title and abstract screening to exclude irrelevant articles based on predefined eligibility criteria, followed by a full-text assessment of the articles using Covidence software. The study selection process is reported in Fig. 1. Any discrepancies in data extraction were resolved through discussion or consultation with a third reviewer.

### Data extraction

Two independent reviewers extracted data from eligible studies using a standardized data collection form. The extracted data included study characteristics [author(s), year of publication, study design, sample size and follow-up duration], patient characteristics (mean age, gender, comorbidities and transplant type), and outcomes. Outcome data included graft survival rates (at 1, 5 and 10 years where available), patient survival rates, incidence of DGF, time to graft function, acute and chronic rejection rates, and causes of graft loss. As graft and patient survival rates were inconsistently reported across studies, crude event numbers were used rather than survival rates to ensure methodological consistency, allowing for a robust pooled analysis using a Mantel–Haenszel method. Extracted data were cross-checked by both reviewers and any discrepancies in data extraction were resolved through discussion or consultation with a third reviewer.

### Risk of bias assessment

The quality of the included studies was assessed using the Newcastle-Ottawa Scale for cohort studies or the Cochrane Risk of Bias tool for randomized controlled trials. Studies were classified as high, moderate or low quality, and only those rated as moderate or high quality were included in the final analysis.

### Statistical analysis

Data analysis was performed using Review Manager (RevMan) software, version 5.4.1 (Nordic Cochrane Centre, The Cochrane Collaboration, 2020, Copenhagen, Denmark), applying the Mantel–Haenszel method to compute pooled odds ratios (ORs) and risk ratios (RRs), when feasible, with 95% confidence intervals (CIs). A random-effects model was applied to account for heterogeneity across studies. The primary outcomes analyzed were graft survival and patient survival.

Sensitivity analyses were conducted to compare survival outcomes at specific time points, including 5-year and 10-year graft and patient survival rates. Additional comparisons were performed between different transplant groups: first KT versus second transplants, second transplants versus third or higher transplants, and third transplants versus fourth transplants.

Secondary outcomes, such as DGF, were also analyzed using ORs with 95% CIs. Forest plots were generated to visually represent pooled estimates and CIs for each outcome. Heterogeneity across studies was evaluated using the I² statistic, with values above 50% considered indicative of substantial heterogeneity. Publication bias was assessed using a funnel plot to identify potential asymmetry, which could suggest reporting bias or small-study effects.

## RESULTS

The initial search across the specified databases identified a total of 705 references. After the removal of duplicates and screening titles and abstracts, 16 studies met the eligibility criteria and were included in the final analysis (Fig. 1) [9, 10, 13–26]. All studies included in the final analysis are summarized in Table 1.

**Figure 2:**
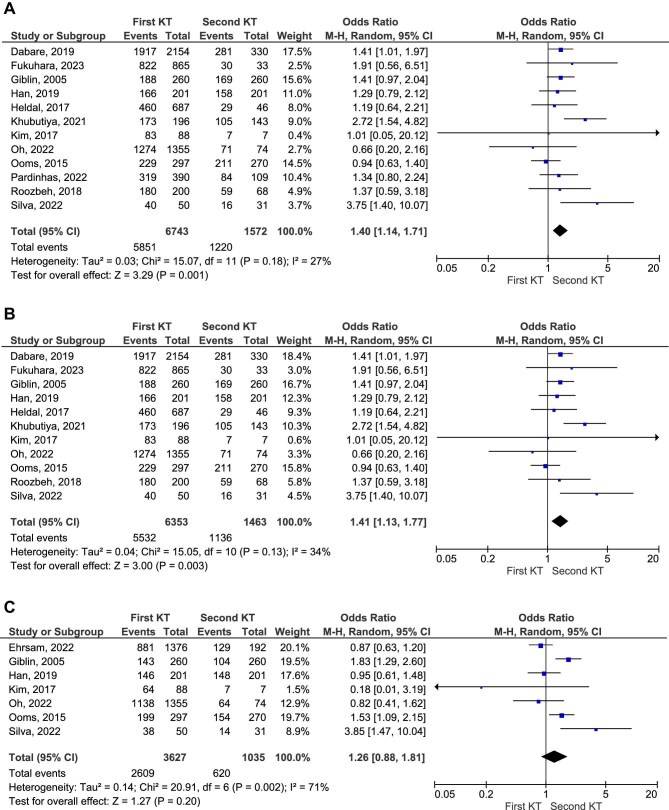
Graft survival in patients who received first KT as compared with those from second transplant group (OR).

Data on long-term graft survival (all years combined) were reported in 12 studies [9, 13–23]. Patients who underwent a first KT had a significantly higher graft survival rate compared with those who received a second transplant (86.7% versus 77.6%), with an OR of 1.40 (95% CI 1.14–1.71, *P* = .001; Fig. 2A; moderate certainty of evidence).

**Figure 3:**
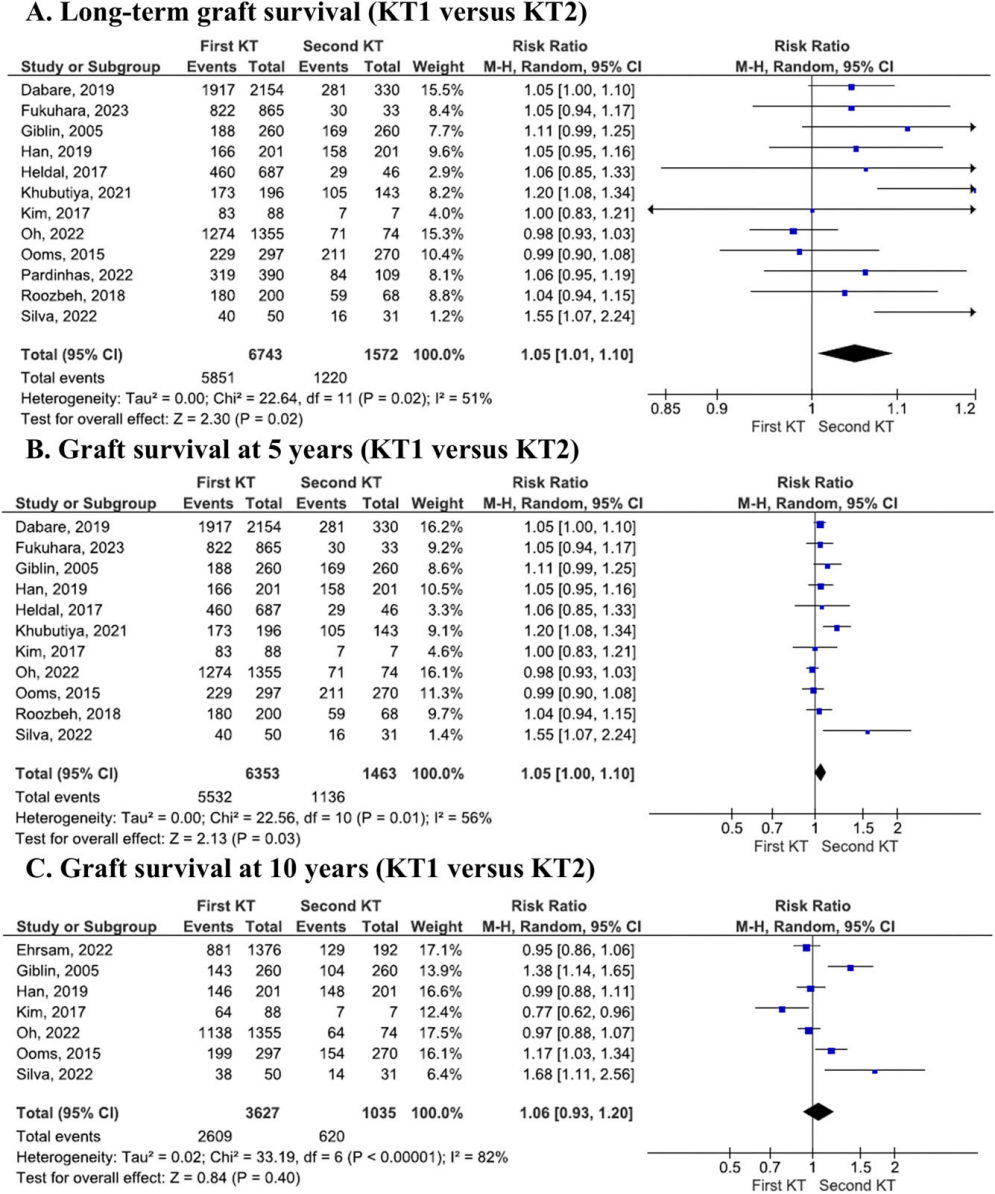
Graft survival in patients who received first KT as compared with those from second transplant group (RR).

Further analyses focused on specific time frames. At 5 years post-transplant, graft survival was significantly better in patients with a first KT compared with those with a second transplant (OR 1.41, 95% CI 1.13–1.77, *P* = .003; Fig. 2B; moderate certainty of evidence). However, 10-year graft survival rates were similar between the two groups (OR 1.26, 95% CI 0.88–1.81, *P* = .20; Fig. 2C; low certainty of evidence). This analysis was limited by high heterogeneity among the included studies. The RR of graft survival rates at each follow-up point are presented in Fig. 3.

**Figure 4:**
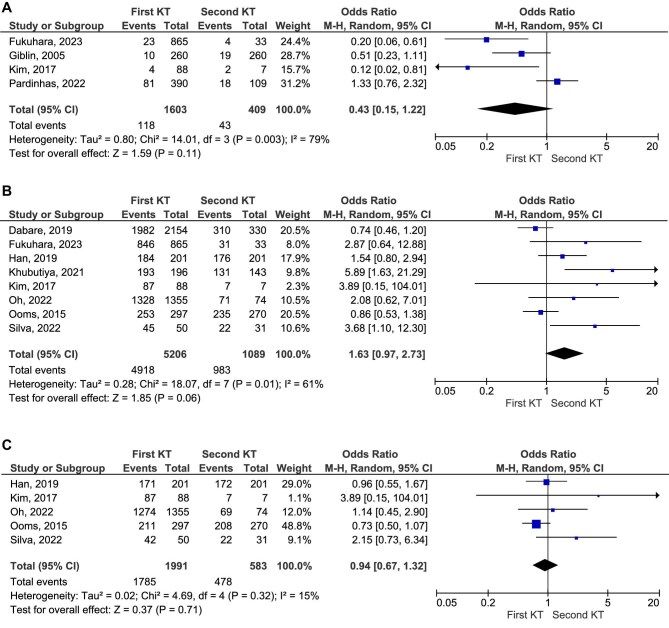
Comparative survival rate of patients who received first and second KT.

While graft survival rates were reported to be higher in first KT recipients in some studies, overall patient survival did not differ significantly between the first and second KT groups (OR 1.25, 95% CI 0.87–1.81, *P* = .23; Fig. 4A; low certainty of evidence). Similarly, no significant differences were found for survival rates at specific time points. The 5-year survival rate comparison showed an OR of 1.63 (95% CI 0.97–2.73, *P* = .06; low certainty of evidence), while the 10-year survival rate analysis resulted in an OR of 0.94 (95% CI 0.67–1.32, *P* = .71; Fig. 4B and C, respectively; low certainty of evidence).

**Figure 5:**
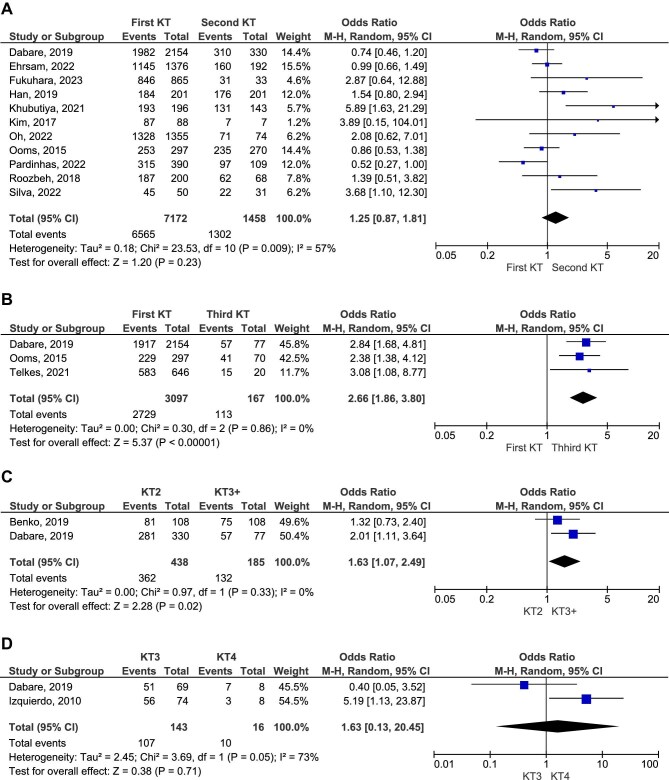
Delayed graft function and graft survival rate at 5 years in different KT patients.

Delayed graft function rates were comparable between first KT recipients and those in the second KT group (*P* = .11; Fig. 5A). Notably, first KT recipients demonstrated 2.66 higher odds of graft survival at 5 years compared with patients who underwent a third transplant (OR 2.66, 95% CI 1.86–3.80, *P* < .00001; Fig. 5B; high certainty of evidence). Additionally, graft survival was significantly better in second KT recipients compared with those who received subsequent retransplants (OR 1.63, 95% CI 1.07–2.49, *P* = .02; Fig. 5C). Meanwhile, third KT recipients exhibited similar graft survival rates to those who underwent a fourth KT (*P* = .71; Fig. 5D).

**Figure 6:**
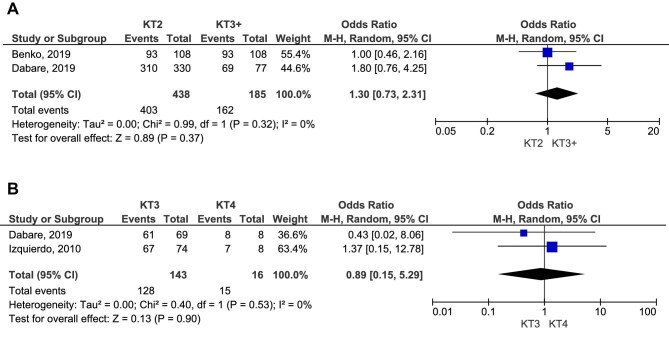
Comparative survival rates in different KT populations.

Second transplant recipients had a similar survival rate to those who underwent subsequent retransplants (OR 1.30, 95% CI 0.73–2.31, *P* = .37; Fig. 6A; moderate certainty of evidence). Likewise, no significant difference was observed in 5-year survival rates between third and fourth KT recipients (OR 0.89, 95% CI 0.15–5.29, *P* = .90; Fig. 6B; low certainty of evidence). However, these findings should be interpreted with caution due to the limited number of studies addressing these outcomes.

**Figure 7:**
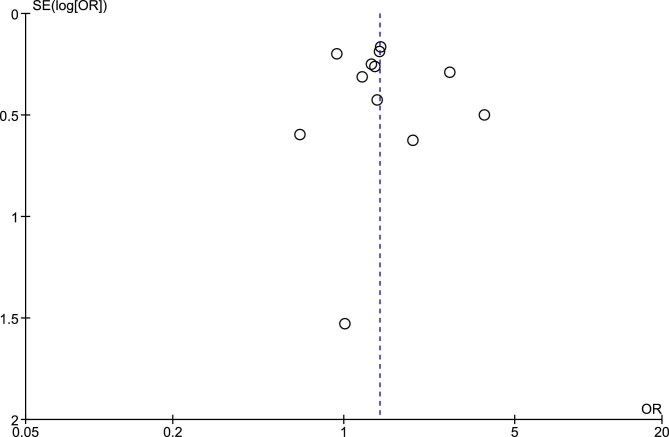
Funnel-plot analysis of included studies.

Publication bias was assessed using funnel plot, as displayed in Fig. 7.

Using the GRADE (Grading of Recommendations Assessment, Development, and Evaluation) approach, we assessed the certainty of evidence across all primary outcomes. Most comparisons between first and second KT had moderate certainty, whereas comparisons involving third or fourth transplants exhibited low certainty due to study heterogeneity and imprecision. These findings should be interpreted with caution, particularly in the context of long-term patient survival.

## DISCUSSION

This meta-analysis, including 16 studies, confirmed that first-time KT recipients generally achieve better survival outcomes compared with those receiving second or subsequent retransplants. At the 5-year mark, first-time transplants demonstrated significantly higher graft survival rates than second transplants (86.7% versus 77.6%, OR 1.40, *P* = .001), underscoring the importance of optimizing conditions for first transplants to maximize outcomes. Interestingly, however, the 10-year graft survival rates between first and second transplant recipients were not significantly different (OR 1.26, 95% CI 0.88–1.81, *P* = .20), suggesting a potential conjunction of outcomes over the long term as factors like chronic allograft dysfunction become prominent across all transplant groups [9]. Adding further insight, a retrospective analysis of paired KT from the same donors further supports these findings, showing no significant differences in graft survival or function between the first and second transplants, even when the second transplant experienced longer cold ischemia times. This controlled setting highlights that while retransplants carry inherent risks, careful management of modifiable factors can mitigate disparities in outcomes, especially in the long-term [27].

There are many factors that could greatly alter the outcomes of second transplants, one of which is the timing of the procedure. Preemptive second KT, performed before the need for dialysis, are associated with significantly better outcomes. Studies show reduced risks of graft loss as well as improved early post-transplant outcomes, including lower rates of acute rejection and DGF [28, 29]. However, these benefits were influenced by prior graft survival, with poorer outcomes observed when the first graft failed within 1 year [29]. These findings underscore the nuanced role of preemptive transplantation, emphasizing its advantages when prior graft survival is favorable while warning against possible risks in less favorable cases.

Although first-time KT recipients showed superior graft survival rates, patient survival did not differ significantly between first and second transplants. The OR for 5-year survival was 1.63 (*P* = .06), suggesting a potential trend favoring first transplants, but this difference was not sustained at 10 years (OR 0.94, *P* = .71). These results not only suggest that patient survival remains unchanged regardless of transplant number but also challenge overly restrictive retransplantation policies. Retransplantation involves a variety of decisions to be made including but not limited to sensitization, comorbidities, and access to organs. However, given the comparable patient survival rates observed in our study, policy adjustments allowing equitable access to retransplantation should be considered. Further research is required to analyze the long-term quality of life and graft outcomes in retransplanted patient groups.

For higher-order retransplants, a pronounced disparity becomes evident; graft survival declined further, with third transplant recipients experiencing more than a 2.5-fold lower graft survival rate at 5 years compared with first-time recipients (OR 2.66, *P* < .00001). Notably, graft survival rates between third and fourth transplants were comparable, suggesting a potential plateau in outcomes for these patients. Patient survival rates also showed no significant differences between second and subsequent transplants (OR 1.30, *P* = .37) or between third and fourth transplants (OR 0.89, *P* = .90). These findings reflect the cumulative immunologic sensitization, heightened rejection risk, and procedural complexity that pose challenges to higher-order transplants.

Our analysis highlights several substantial implications for clinical practice and policy. The marked advantage of first-time transplants underscores the need to optimize initial graft survival through intensive pre-transplant preparation, enhanced immunosuppression protocols, rigorous monitoring and patient education [2, 30, 31]. The relatively favorable outcomes for second transplants, particularly in the long term, support the prioritization of retransplantation over dialysis in eligible patients. However, the diminishing benefits seen with higher-order retransplants call for careful patient selection and tailored management strategies to moderate risks and improve outcomes. Furthermore, the comparable survival rates in third and fourth transplant recipients suggest that with appropriate immunological and perioperative care, these procedures remain a viable option for selected patients.

Despite the insights provided by this study, several limitations must be acknowledged. A limitation of this meta-analysis is the inability to account for potential confounding factors, such as donor and recipient characteristics, ischemia time and immunosuppression regimens. While some of the included studies reported adjusted effect measures, the heterogeneity in adjustment models and covariates prevented us from pooling these estimates in a meta-analysis. As a result, our findings may be influenced by residual confounding, and caution is warranted in their interpretation. High heterogeneity was observed in the analysis of 10-year graft survival rates, likely due to variations in study designs, patient demographics and treatment protocols. Furthermore, the limited number of studies addressing third and fourth transplants restricts the generalizability of findings in these subgroups. This underscores the need for more comprehensive research to better understand outcomes for patients undergoing multiple retransplants. Long-term cohort studies could also provide valuable insights into survival trends beyond the 10-year mark and clarify specific causes of graft failure and patient mortality in these populations. Additionally, selection bias is an inherent limitation of retransplantation studies, as patient who survive long enough to receive a second or third transplant may have a better baseline health status than those who do not. Furthermore, variations in waiting times for retransplantation introduce immortal time bias, as patients who experience graft failure before receiving a second KT are not accounted in the analysis. Future studies should implement time-dependent methods to mitigate these biases.

## CONCLUSION

This meta-analysis highlights the superior graft survival rates for first-time KT, with outcomes for second transplants showing comparable long-term survival despite initial post-transplant challenges. Moving forward, further research focused on higher-order retransplants, long-term graft failure mechanisms, and the role of preemptive retransplantation in optimizing outcomes will be essential for refining clinical practices and improving survival for this complex patient group.

## Supplementary Material

gfaf066_Supplemental_Files

## Data Availability

No new data were generated or analyzed in support of this research.
