# Supplementary material for: A meta-analysis of graft survival, patient survival and delayed graft function in first-time and repeat kidney transplants
Source: Nephrol Dial Transplant. 2025 Apr 15;40(10):1906–18. doi: 10.1093/ndt/gfaf066 (PMC12477473; doi:10.1093/ndt/gfaf066)
Supplement: gfaf066_Supplemental_Files [file gfaf066_supplemental_files.zip › 130 Supplementary_Table_S1_(2)_(1).docx]

**Supplementary Table S1**. Search strategy and keywords for systematic database search

| **Total Records: n= 705** |
| --- |
| **PubMed (n=72)** |
| **PubMed**  3 ("first kidney transplantation"[tiab:~2] OR "first kidney transplant*" OR "first renal Transplant*" OR "first renal transplantation"[tiab:~2] OR "first Kidney Graft*" OR "first kidney grafting"[tiab:~2] OR "1th kidney transplantation"[tiab:~2] OR "1th renal transplantation"[tiab:~2] OR "1th kidney grafting"[tiab:~2]) AND ("second kidney transplantation"[tiab:~2] OR "second kidney transplant*" OR "second renal Transplant*" OR "second renal transplantation"[tiab:~2] OR "second kidney graft*" OR "second kidney grafting"[tiab:~2] OR "2nd kidney transplantation"[tiab:~2] OR "2nd renal transplantation"[tiab:~2] OR "2nd kidney grafting"[tiab:~2] OR "third kidney transplantation"[tiab:~2] OR "third kidney transplant*" OR "third renal Transplant*" OR "third renal transplantation"[tiab:~2] OR "third kidney grafting"[tiab:~2] OR "3rd kidney transplantation"[tiab:~2] OR "3rd renal transplantation"[tiab:~2] OR "3rd kidney grafting"[tiab:~2]) Most Recent ("first kidney transplantation"[Title/Abstract:~2] OR "first kidney transplant*"[All Fields] OR "first renal transplant*"[All Fields] OR "first renal transplantation"[Title/Abstract:~2] OR "first kidney graft*"[All Fields] OR "first kidney grafting"[Title/Abstract:~2] OR "1th kidney transplantation"[Title/Abstract:~2] OR "1th renal transplantation"[Title/Abstract:~2] OR "1th kidney grafting"[Title/Abstract:~2]) AND ("second kidney transplantation"[Title/Abstract:~2] OR "second kidney transplant*"[All Fields] OR "second renal transplant*"[All Fields] OR "second renal transplantation"[Title/Abstract:~2] OR "second kidney graft*"[All Fields] OR "second kidney grafting"[Title/Abstract:~2] OR "2nd kidney transplantation"[Title/Abstract:~2] OR "2nd renal transplantation"[Title/Abstract:~2] OR "2nd kidney grafting"[Title/Abstract:~2] OR "third kidney transplantation"[Title/Abstract:~2] OR "third kidney transplant*"[All Fields] OR "third renal transplant*"[All Fields] OR "third renal transplantation"[Title/Abstract:~2] OR "third kidney grafting"[Title/Abstract:~2] OR "3rd kidney transplantation"[Title/Abstract:~2] OR "3rd renal transplantation"[Title/Abstract:~2] OR "3rd kidney grafting"[Title/Abstract:~2]) 72  2 "second kidney transplantation"[tiab:~2] OR "second kidney transplant*" OR "second renal Transplant*" OR "second renal transplantation"[tiab:~2] OR "second kidney graft*" OR "second kidney grafting"[tiab:~2] OR "2nd kidney transplantation"[tiab:~2] OR "2nd renal transplantation"[tiab:~2] OR "2nd kidney grafting"[tiab:~2] OR "third kidney transplantation"[tiab:~2] OR "third kidney transplant*" OR "third renal Transplant*" OR "third renal transplantation"[tiab:~2] OR "third kidney grafting"[tiab:~2] OR "3rd kidney transplantation"[tiab:~2] OR "3rd renal transplantation"[tiab:~2] OR "3rd kidney grafting"[tiab:~2] Most Recent "second kidney transplantation"[Title/Abstract:~2] OR "second kidney transplant*"[All Fields] OR "second renal transplant*"[All Fields] OR "second renal transplantation"[Title/Abstract:~2] OR "second kidney graft*"[All Fields] OR "second kidney grafting"[Title/Abstract:~2] OR "2nd kidney transplantation"[Title/Abstract:~2] OR "2nd renal transplantation"[Title/Abstract:~2] OR "2nd kidney grafting"[Title/Abstract:~2] OR "third kidney transplantation"[Title/Abstract:~2] OR "third kidney transplant*"[All Fields] OR "third renal transplant*"[All Fields] OR "third renal transplantation"[Title/Abstract:~2] OR "third kidney grafting"[Title/Abstract:~2] OR "3rd kidney transplantation"[Title/Abstract:~2] OR "3rd renal transplantation"[Title/Abstract:~2] OR "3rd kidney grafting"[Title/Abstract:~2] 507  1 "first kidney transplantation"[tiab:~2] OR "first kidney transplant*" OR "first renal Transplant*" OR "first renal transplantation"[tiab:~2] OR "first Kidney Graft*" OR "first kidney grafting"[tiab:~2] OR "1th kidney transplantation"[tiab:~2] OR "1th renal transplantation"[tiab:~2] OR "1th kidney grafting"[tiab:~2] Most Recent "first kidney transplantation"[Title/Abstract:~2] OR "first kidney transplant*"[All Fields] OR "first renal transplant*"[All Fields] OR "first renal transplantation"[Title/Abstract:~2] OR "first kidney graft*"[All Fields] OR "first kidney grafting"[Title/Abstract:~2] OR "1th kidney transplantation"[Title/Abstract:~2] OR "1th renal transplantation"[Title/Abstract:~2] OR "1th kidney grafting"[Title/Abstract:~2] 1,388 |
| **Scopus (n=55)** |
| TITLE-ABS-KEY ( "first w/2 kidney transplantation" OR "first kidney transplant*" OR "first renal transplant*" OR "first w/2 renal transplantation" OR "first Kidney Graft*" OR "first w/2 kidney grafting" OR "1th w/2 kidney transplantation" OR "1th w/2 renal transplantation" OR "1th w/2 kidney grafting" ) AND TITLE-ABS-KEY ( "second w/2 kidney transplantation" OR "second kidney transplant*" OR "second renal transplant*" OR "second w/2 renal transplantation" OR "second kidney graft*" OR "second w/2 kidney grafting" OR "2nd w/2 kidney transplantation" OR "2nd w/2 renal transplantation" OR "2nd w/2 kidney grafting" OR "third w/2 kidney transplantation" OR "third kidney transplant*" OR "third renal Transplant*" OR "third w/2 renal transplantation" OR "third w/2 kidney grafting" OR "3rd w/2 kidney transplantation" OR "3rd w/2 renal transplantation" OR "3rd w/2 kidney grafting" ) |
| **Web of Science (n=50)** |
| **Web of Science**  1: ALL=("first NEAR/2 kidney transplantation" OR "first kidney transplant*" OR "first renal transplant*" OR "first NEAR/2 renal transplantation" OR "first Kidney Graft*" OR "first NEAR/2 kidney grafting" OR "1th NEAR/2 kidney transplantation" OR "1th NEAR/2 renal transplantation" OR "1th NEAR/2 kidney grafting") Date Run: Mon Oct 07 2024 17:12:01 GMT+0300 (GMT+03:00) Results: 585  2: ALL=("second NEAR/2 kidney transplantation" OR "second kidney transplant*" OR "second renal transplant*" OR "second NEAR/2 renal transplantation" OR "second kidney graft*" OR "second NEAR/2 kidney grafting" OR "2nd NEAR/2 kidney transplantation" OR "2nd NEAR/2 renal transplantation" OR "2nd NEAR/2 kidney grafting" OR "third NEAR/2 kidney transplantation" OR "third kidney transplant*" OR "third renal transplant*" OR "third NEAR/2 renal transplantation" OR "third NEAR/2 kidney grafting" OR "3rd NEAR/2 kidney transplantation" OR "3rd NEAR/2 renal transplantation" OR "3rd NEAR/2 kidney grafting") Date Run: Mon Oct 07 2024 17:13:58 GMT+0300 (GMT+03:00) Results: 389  3: #2 AND #1 Date Run: Mon Oct 07 2024 17:14:28 GMT+0300 (GMT+03:00) Results: 50 |
| **Cochrane Library n=470** |
| "first/2 kidney transplantation" OR first kidney NEXT transplant* OR first renal NEXT transplant* OR "first/2 renal transplantation" OR first Kidney NEXT Graft* OR "first/2 kidney grafting" OR "1th/2 kidney transplantation" OR "1th/2 renal transplantation" OR "1th/2 kidney grafting" in Title Abstract Keyword AND "second/2 kidney transplantation" OR second kidney NEXT transplant* OR second renal NEXT transplant* OR "second/2 renal transplantation" OR second kidney NEXT graft* OR "second/2 kidney grafting" OR "2nd/2 kidney transplantation" OR "2nd/2 renal transplantation" OR "2nd/2 kidney grafting" OR "third/2 kidney transplantation" OR third kidney NEXT transplant* OR third renal NEXT Transplant* OR "third/2 renal transplantation" OR "third/2 kidney grafting" OR "3rd/2 kidney transplantation" OR "3rd/2 renal transplantation" OR "3rd/2 kidney grafting" in Title Abstract Keyword - (Word variations have been searched) |
| **Ovid MEDLINE (n=58)** |
| **Ovid MEDLINE(R) and Epub Ahead of Print, In-Process, In-Data-Review & Other Non-Indexed Citations, Daily and Versions <1946 to October 04, 2024>**  1 (first adj2 kidney transplantation).mp. [mp=title, book title, abstract, original title, name of substance word, subject heading word, floating sub-heading word, keyword heading word, organism supplementary concept word, protocol supplementary concept word, rare disease supplementary concept word, unique identifier, synonyms, population supplementary concept word, anatomy supplementary concept word] 234  2 "first kidney transplant*".mp. 399  3 "first renal transplant*".mp. 197  4 (first adj2 renal transplantation).mp. [mp=title, book title, abstract, original title, name of substance word, subject heading word, floating sub-heading word, keyword heading word, organism supplementary concept word, protocol supplementary concept word, rare disease supplementary concept word, unique identifier, synonyms, population supplementary concept word, anatomy supplementary concept word] 117  5 "first Kidney Graft*".mp. 46  6 (first adj2 kidney grafting).mp. [mp=title, book title, abstract, original title, name of substance word, subject heading word, floating sub-heading word, keyword heading word, organism supplementary concept word, protocol supplementary concept word, rare disease supplementary concept word, unique identifier, synonyms, population supplementary concept word, anatomy supplementary concept word] 0  7 (1th adj2 kidney transplantation).mp. [mp=title, book title, abstract, original title, name of substance word, subject heading word, floating sub-heading word, keyword heading word, organism supplementary concept word, protocol supplementary concept word, rare disease supplementary concept word, unique identifier, synonyms, population supplementary concept word, anatomy supplementary concept word] 0  8 (1th adj2 renal transplantation).mp. [mp=title, book title, abstract, original title, name of substance word, subject heading word, floating sub-heading word, keyword heading word, organism supplementary concept word, protocol supplementary concept word, rare disease supplementary concept word, unique identifier, synonyms, population supplementary concept word, anatomy supplementary concept word] 0  9 (1th adj2 kidney grafting).mp. [mp=title, book title, abstract, original title, name of substance word, subject heading word, floating sub-heading word, keyword heading word, organism supplementary concept word, protocol supplementary concept word, rare disease supplementary concept word, unique identifier, synonyms, population supplementary concept word, anatomy supplementary concept word] 0  10 1 or 2 or 3 or 4 or 5 or 6 or 7 or 8 or 9 812  11 (second adj2 kidney transplantation).mp. [mp=title, book title, abstract, original title, name of substance word, subject heading word, floating sub-heading word, keyword heading word, organism supplementary concept word, protocol supplementary concept word, rare disease supplementary concept word, unique identifier, synonyms, population supplementary concept word, anatomy supplementary concept word] 92  12 "second kidney transplant*".mp. 170  13 "second renal transplant*".mp. 136  14 (second adj2 renal transplantation).mp. [mp=title, book title, abstract, original title, name of substance word, subject heading word, floating sub-heading word, keyword heading word, organism supplementary concept word, protocol supplementary concept word, rare disease supplementary concept word, unique identifier, synonyms, population supplementary concept word, anatomy supplementary concept word] 59  15 "second kidney graft*".mp. 30  16 (second adj2 kidney grafting).mp. [mp=title, book title, abstract, original title, name of substance word, subject heading word, floating sub-heading word, keyword heading word, organism supplementary concept word, protocol supplementary concept word, rare disease supplementary concept word, unique identifier, synonyms, population supplementary concept word, anatomy supplementary concept word] 0  17 (2nd adj2 kidney transplantation).mp. [mp=title, book title, abstract, original title, name of substance word, subject heading word, floating sub-heading word, keyword heading word, organism supplementary concept word, protocol supplementary concept word, rare disease supplementary concept word, unique identifier, synonyms, population supplementary concept word, anatomy supplementary concept word] 2  18 (2nd adj2 renal transplantation).mp. [mp=title, book title, abstract, original title, name of substance word, subject heading word, floating sub-heading word, keyword heading word, organism supplementary concept word, protocol supplementary concept word, rare disease supplementary concept word, unique identifier, synonyms, population supplementary concept word, anatomy supplementary concept word] 1  19 (2nd adj2 kidney grafting).mp. [mp=title, book title, abstract, original title, name of substance word, subject heading word, floating sub-heading word, keyword heading word, organism supplementary concept word, protocol supplementary concept word, rare disease supplementary concept word, unique identifier, synonyms, population supplementary concept word, anatomy supplementary concept word] 0  20 (third adj2 kidney transplantation).mp. [mp=title, book title, abstract, original title, name of substance word, subject heading word, floating sub-heading word, keyword heading word, organism supplementary concept word, protocol supplementary concept word, rare disease supplementary concept word, unique identifier, synonyms, population supplementary concept word, anatomy supplementary concept word] 21  21 "third kidney transplant*".mp. 40  22 "third renal Transplant*".mp. 27  23 (third adj2 renal transplantation).mp. [mp=title, book title, abstract, original title, name of substance word, subject heading word, floating sub-heading word, keyword heading word, organism supplementary concept word, protocol supplementary concept word, rare disease supplementary concept word, unique identifier, synonyms, population supplementary concept word, anatomy supplementary concept word] 15  24 (third adj2 kidney grafting).mp. [mp=title, book title, abstract, original title, name of substance word, subject heading word, floating sub-heading word, keyword heading word, organism supplementary concept word, protocol supplementary concept word, rare disease supplementary concept word, unique identifier, synonyms, population supplementary concept word, anatomy supplementary concept word] 0  25 (3rd adj2 kidney transplantation).mp. [mp=title, book title, abstract, original title, name of substance word, subject heading word, floating sub-heading word, keyword heading word, organism supplementary concept word, protocol supplementary concept word, rare disease supplementary concept word, unique identifier, synonyms, population supplementary concept word, anatomy supplementary concept word] 0  26 (3rd adj2 renal transplantation).mp. [mp=title, book title, abstract, original title, name of substance word, subject heading word, floating sub-heading word, keyword heading word, organism supplementary concept word, protocol supplementary concept word, rare disease supplementary concept word, unique identifier, synonyms, population supplementary concept word, anatomy supplementary concept word] 0  27 (3rd adj2 kidney grafting).mp. [mp=title, book title, abstract, original title, name of substance word, subject heading word, floating sub-heading word, keyword heading word, organism supplementary concept word, protocol supplementary concept word, rare disease supplementary concept word, unique identifier, synonyms, population supplementary concept word, anatomy supplementary concept word] 0  28 11 or 12 or 13 or 14 or 15 or 16 or 17 or 18 or 19 or 20 or 21 or 22 or 23 or 24 or 25 or 26 or 27 425  29 10 and 28 58 |
