# Supplementary material for: A meta-analysis of graft survival, patient survival and delayed graft function in first-time and repeat kidney transplants
Source: Nephrol Dial Transplant. 2025 Apr 15;40(10):1906–18. doi: 10.1093/ndt/gfaf066 (PMC12477473; doi:10.1093/ndt/gfaf066)
Supplement: gfaf066_Supplemental_Files [file gfaf066_supplemental_files.zip › 130 Supplementary_Table_S2_and_S3..docx]

**Supplementary Table S2: Quality assessment of included studies using the Newcastle-Ottawa Scale for cohort studies**

| **Study** | **Study**  **Design** | **Selection** | | | | **Comparability** | **Outcome** | | | **Total  Score** |
| --- | --- | --- | --- | --- | --- | --- | --- | --- | --- | --- |
|  |  | *Representative of exposed cohort* | *Selection of non-exposed cohort* | *Ascertainment of exposure* | *Outcome not present at start* | *On the basis of design or analysis* | *Assessment of outcome* | *Follow-up >1 year* | *Follow-up adequate* |  |
| Benko (2019) | Retrospective Cohort | * | * | * | * | ** | * | * |  | **8** |
| Dabare (2019) | Retrospective Cohort | * | * | * | * |  | * | * | * | **7** |
| Ehrsam (2022) | Retrospective cohort | * | * | * | * | ** | * | * | * | **9** |
| Fukuhara (2023) | Retrospective Cohort | * | * | * | * | ** | * | * |  | **8** |
| Giblin (2005) | Retrospective Cohort | * | * | * | * | ** | * | * |  | **8** |
| Han (2019) | Retrospective Cohort | * | * | * | * | ** | * | * |  | **8** |
| Heldal (2017) | Retrospective Cohort |  | * | * | * |  | * | * |  | **5** |
| Izquierdo (2005) | Retrospective Cohort | * | * | * | * |  | * | * |  | **6** |
| Khubutiya (2021) | Retrospective Cohort | * | * | * | * |  | * |  |  | **5** |
| Kim (2017) | Retrospective Cohort |  | * | * | * |  | * | * | * | **6** |
| Oh (2022) | Retrospective Cohort | * | * | * | * |  | * | * |  | **6** |
| Pardinhas (2022) | Retrospective Cohort | * | * | * | * |  | * | * |  | **6** |
| Roozbeh (2018) | Retrospective Cohort | * | * | * | * |  | * | * | * | **7** |
| Silva (2022) | Retrospective Cohort | * | * | * | * |  | * | * | * | **7** |
| Telkes (2021) | Retrospective Cohort | * | * | * | * | ** | * | * |  | **8** |

The grading of the studies is conducted according to the scale and coding manual.^1^

^1^Wells, G. A., Shea, B., O'Connell, D., Peterson, J., Welch, V., Losos, M., & Tugwell, P. (2014). The Newcastle-Ottawa Scale (NOS) for assessing the quality of nonrandomized studies in meta-analyses. Ottawa Hospital Research Institute.

**Supplementary Table S3. Quality assessment of included studies using the Newcastle-Ottawa Scale for case-control studies**

| **Study** | **Selection** | | | | **Comparability** | **Exposure** | | | **Total  Score** |
| --- | --- | --- | --- | --- | --- | --- | --- | --- | --- |
|  | *Case definition adequate* | *Representativeness of the cases* | *Selection of Controls* | *Definition of Controls* | *On the basis of design or analysis* | *Ascertainment of exposure* | *Same method of ascertainment for cases and controls* | *Non-Response rate* |  |
| Ooms (2015) | * |  | * | * | ** | * | * |  | **7** |

The grading of the studies is conducted according to the scale and coding manual.^1^

^1^Wells, G. A., Shea, B., O'Connell, D., Peterson, J., Welch, V., Losos, M., & Tugwell, P. (2014). The Newcastle-Ottawa Scale (NOS) for assessing the quality of nonrandomized studies in meta-analyses. Ottawa Hospital Research Institute.
